# Neurobiological Mechanisms of Acupuncture

**DOI:** 10.1155/2013/652457

**Published:** 2013-11-28

**Authors:** Lijun Bai, Richard E. Harris, Jian Kong, Lixing Lao, Vitaly Napadow, Baixiao Zhao

**Affiliations:** ^1^The Key Laboratory of Biomedical Information Engineering, Ministry of Education, Department of Biomedical Engineering, School of Life Science and Technology, Xi'an Jiaotong University, Xi'an 710049, China; ^2^Department of Anesthesiology, University of Michigan, Ann Arbor, MI 48109, USA; ^3^Department of Psychiatry, Massachusetts General Hospital (MGH), Harvard Medical School, Boston, MA 02129, USA; ^4^School of Chinese Medicine, The University of Hong Kong, 10 Sassoon Road, Pokfulam, Hong Kong; ^5^MGH/HMS/MIT Martinos Center for Biomedical Imaging, Massachusetts General Hospital, Harvard Medical School, Boston, MA 02129, USA; ^6^School of Acupuncture-Moxibustion and Tuina, Beijing University of Chinese Medicine, Beijing 100029, China

Acupuncture, an age-old healing art, has been accepted to effectively treat various diseases, particularly chronic pain and neurodegenerative diseases. Since its public acceptance and good efficacy, increasing attention has been now paid to exploring the physiological and biochemical mechanisms underlying acupuncture, particularly the brain mechanisms. Basic and clinical acupuncture studies on neurobiological mechanisms of acupuncture are crucial for the development of acupuncture. This issue compiles 32 exciting papers, most of which are very novel and excellent investigations in this field.

The neural mechanism underlying acupuncture analgesia was addressed in four articles. J. Wang et al. established a postincisional pain model of rats and investigated electroacupuncture effect on the brain oscillations involving postoperative pain. B.-S. Lim et al., using interesting bee venom acupuncture, explored whether it can relieve oxaliplatin-induced cold allodynia and which endogenous analgesic system is implicated. The paper by Yumi Maeda et al. aimed to evaluate this linkage between brain response to acupuncture and subsequent analgesia in chronic pain patients with carpal tunnel syndrome. Since many articles aimed to assess the effect of electroacupuncture induced analgesia, W. Kim et al. systemically conducted a review to assess the efficacy and clarify its mechanism on neuropathic pain. In addition, as a great challenge in acupuncture analgesia and treatment evaluations, D. Zhu et al. outlined the advantages and disadvantages of kinds of acupuncture controls and highlighted how the differences among placebo devices can be used to isolate distinct components of acupuncture treatment.

Five papers deal with potential neural mechanism underlying acupuncture treatments on the stroke, hypertension, and mild cognitive impairments. The paper by L. Liu and R.T.F. Cheung aimed to investigate whether the combination of melatonin and electroacupuncture therapies could be beneficial against transient focal cerebral ischemia in a rat model of transient middle cerebral artery occlusion. W. Qin et al. accompanied by L. Liu and R.T.F. Cheung's paper explored the importance of anti-inflammatory acupuncture treatment for the focal cerebral ischemia/reperfusion by inhibiting the NF-*κ*B signaling pathway. Another two papers mainly focused on the acupuncture modulation of neural mechanism for hypertension regulation with long-term as well as short-term treatments. The paper by G.-H. Tian et al. addressed the long-term electroacupuncture on cerebral microvessels and neurons in CA1 region of hippocampus in spontaneously hypertensive rats. H. Chen et al. aimed to explore the hypothalamus-anchored resting brain network underlying primary hypertension patients after short-term acupuncture treatments. Moreover, for mild cognitive impairments, S. Chen et al. have pointed out that acupuncture at KI3 at different cognitive states and with varying needling depths may induce distinct reorganizations of effective connectivity of brain networks.

The neuroendocrine system involving acupuncture has been addressed by the following three papers. Z. Yu et al. has suggested that TRPV1 receptor is partially involved in the electroacupuncture-mediated modulation of gastric motility. The paper by C.-C. Kuo et al. explored the mechanism of electroacupuncture (EAc) induced antinociception involved opioid receptors and the serotonergic system. Q.-Q. Li et al. conducted a systemic review about the central mechanism of acupuncture in modulating various autonomic responses. Moreover, other papers focused on the relatively acupoint specificity from wide aspects. One of the studies by L. Li et al. suggested that both the size and function of the acupoints comply with the functionality of the internal organs; thus the sensitive degree of acupoints changed according to malfunction of internal organs. C.-Y. Chen et al. implied that somatoparasympathetic neuronal connection (groin-spinal dorsal horn-NTS/DMX-uterus) and a somatosympathetic neuronal connection (groin-spinal dorsal horn-NTS-PVN-uterus) could be the prerequisites to the neuronal basis of the somatovisceral reflex and also the neuronal mechanism of acupuncture. Z. Wang et al. advanced that the modulatory effects of different needling sensations induced by relatively different acupoints contribute to acupuncture modulations of limbic-paralimbic-neocortical network. Although the acupoints were spatially adjacent, there was also relatively functional specificity inflected by brain networks.

By gathering these papers, we hope to enrich our readers and researchers with respect to the underlying neurological mechanism of acupuncture, and we look forward to an increasing number of both clinical trials and experimental studies to further promote the development of understanding the neurological mechanism involving acupuncture.

**Lijun  Bai** **Lijun  Bai** **Richard  E.  Harris** **Richard  E.  Harris** **Jian  Kong** **Jian  Kong** **Lixing  Lao** **Lixing  Lao** **Vitaly  Napadow** **Vitaly  Napadow** **Baixiao  Zhao** **Baixiao  Zhao**

